# Historical Review of the Use of Relative Risk Statistics in the Portrayal of the Purported Hazards of High LDL Cholesterol and the Benefits of Lipid-Lowering Therapy

**DOI:** 10.7759/cureus.38391

**Published:** 2023-05-01

**Authors:** David M Diamond, Paul E Leaverton

**Affiliations:** 1 Psychology, University of South Florida, Tampa, USA; 2 Epidemiology and Biostatistics, University of South Florida, Tampa, USA

**Keywords:** statistics, statins, hypercholesterolemia, absolute risk, relative risk

## Abstract

The manner in which clinical trial investigators present their findings to healthcare providers and the public can have a substantial influence on their impact. For example, if a heart attack occurs in 2% of those in the placebo group and in 1% of those in the drug-treated group, the benefit to the treated population is only one percentage point better than no treatment. This finding is unlikely to generate much enthusiasm from the study sponsors and in the reporting of the findings to the public. Instead, trial directors can amplify the magnitude of the appearance of the treatment benefit by using the relative risk (RR) value of a 50% reduction of the risk of a heart attack, since one is 50% of two. By using the RR type of data analysis, clinical trial directors can promote the outcome of their trial in their publication and to the media as highly successful while minimizing or disregarding entirely the absolute risk (AR) reduction of only one percentage point. The practice of expressing the RR without the AR has become routinely deployed in the reporting of findings in many different areas of clinical research. We have provided a historical perspective on how this form of data presentation has become commonplace in the reporting of findings from randomized controlled trials (RCTs) on coronary heart disease (CHD) event monitoring and prevention over the past four decades. We assert that the emphasis on RR coupled with insufficient disclosure of AR in the reporting of RCT outcomes has led healthcare providers and the public to overestimate concerns about high cholesterol and to be misled as to the magnitude of the benefits of cholesterol-lowering therapy. The goal of this review is to prompt the scientific community to address this misleading approach to data presentation.

## Introduction and background

Richard Horton, editor of the Lancet, expressed the opinion that “much of the scientific literature, perhaps half, may simply be untrue” [1]. A similar sentiment was expressed by John Ioannidis, professor of medicine, epidemiology, and population health at Stanford, who stated, “There is increasing concern that in modern research, false findings may be the majority or even the vast majority of published research” [2]. Marcia Angell, former editor of The New England Journal of Medicine (NEJM), disclosed, “It is simply no longer possible to believe much of the clinical research that is published, or to rely on the judgment of trusted physicians or authoritative medical guidelines” [3]. The skepticism over the credibility of much of the published research may have occurred, in part, from decades of misleading presentations of research findings by clinical trial directors.

While these eminent leaders of medical establishments have lamented over a range of flaws in the conduct of medical research, we have focused on one aspect of data analysis that has been deployed by many trial directors to promote their agenda, rather than to present their findings in the most objective manner possible. Specifically, directors have deployed a statistical strategy that can amplify a modest benefit of drug treatment to appear as if the effect is of great clinical significance. This statistical strategy focuses on the use of relative risk (RR) reduction and the exclusion or minimization of absolute risk (AR) reduction, which are two different ways to express the same raw data. The data format and clinical relevance of this issue were addressed by Meleady and Graham [4] over two decades ago: “If an 80 per cent mortality occurs in the placebo group and 40 per cent in the treatment group, this intervention has a 50 per cent relative mortality reduction and a 40 per cent absolute mortality reduction, which is both clinically and statistically important. If 2 per cent mortality occurs in the placebo and 1 per cent in the treatment group, again a 50 per cent relative mortality reduction has occurred but the absolute mortality reduction is 1 per cent, a clinically trivial reduction.”

Skolbekken [5] also illustrated this issue with a more extreme example in his critique of how the benefits of cholesterol-lowering therapy had been portrayed. The following is our summary of the data from hypothetical studies in his Table 1: In one randomized controlled trial (RCT), 2,000 people die out of 10,000 in a placebo group, and 1,000 people die out of 10,000 in a treated group, resulting in an AR reduction of 10% (1,000/10,000=10%). In another RCT, two people die out of 10,000 in a placebo group, and one person dies out of 10,000 in a treated group, resulting in an AR reduction of 0.01% (1/10,000=0.01%). Despite the vast difference in the ARs between the two studies (10% and 0.01%), in both, the RR reduction was 50% (1,000 is 50% of 2,000, and one is 50% of two).

Skolbekken asserted that the “real impact of treatment … can only be seen by also reviewing the absolute risk reduction.” He also noted that reporting only the relative risk gives “a more favourable impression of the effectiveness of a drug than absolute risk estimates.” Numerous investigators have emphasized the importance of this issue; surveys have shown that lay people, as well as healthcare providers, overestimate the benefit of a treatment, such as cholesterol reduction, when the findings are presented solely as the RR [6-16].

In this review, we have provided a historical perspective on the miscommunication of risk and treatment benefit in cholesterol-heart disease research with an in-depth analysis of the following five landmark randomized controlled trials (RCTs) that assessed coronary event monitoring and prevention over the past four decades: 1984, “Lipid Research Clinics Coronary Primary Prevention Trial” (LRC-CPPT) [17,18]; 1986, “Multiple Risk Factor Intervention Trial” (MRFIT) [19]; 2008, “Justification for the Use of Statins in Prevention: An Intervention Trial Evaluating Rosuvastatin” (JUPITER) [20]; 2017, “Further Cardiovascular Outcomes Research with PCSK9 Inhibition in Subjects with Elevated Risk” (FOURIER) [21]; and 2023, “Cholesterol Lowering via Bempedoic Acid [ECT1002], an ACL-Inhibiting Regimen” (CLEAR) [22].

These five clinical trials have been selected because they played a key role in implicating high total serum cholesterol, in general, and low-density lipoprotein cholesterol (LDL-C), in particular, in causing heart disease and in promoting the pharmacological reduction of cholesterol to prevent coronary heart disease (CHD). Our review addresses how the findings in each of these clinical trials were presented to all audiences primarily in the RR format, which exaggerated the role of cholesterol in CHD and amplified the modest benefits of lipid reduction.

## Review

LRC-CPPT: Miscommunication of the benefits of lipid-lowering therapy

LRC-CPPT addressed the hypothesis that “long-term reduction of serum cholesterol in hypercholesterolemic men initially free of CHD will lead to a lowered incidence of coronary heart disease” [17]. Approximately half a million middle-aged males were screened to find those with the highest (top 5%) cholesterol levels (n=3,806). These hypercholesterolemic males were put on a low-cholesterol diet for 7.4 years. In addition, about half of the males were given a placebo (n=1,900), and the remainder (n=1906) were given a bile-sequestering agent (cholestyramine), which reduced cholesterol levels [18].

The authors reported that cholesterol reduction resulted in “a 24% reduction in definite CHD death and a 19% reduction in nonfatal myocardial infarction.” The investigators emphasized the importance and clinical relevance of their findings by stating, “The consistency of the reductions in CHD manifestations observed with cholestyramine in this controlled trial … leaves little doubt of the benefit of cholestyramine therapy” [18].

There was widespread praise for the LRC-CPPT findings, as exemplified by an editorial in the British Medical Journal (BMJ), which stated, “At long last we have clear evidence that reducing very high plasma concentrations of cholesterol and low density lipoprotein (LDL) cholesterol lowers the incidence of coronary heart disease” [23]. Similar views were expressed in a lead article in the Medical Journal of Australia entitled “The lipid hypothesis is proven,” which declared “the incidence of death from definite CHD was reduced by 24% in the cholestyramine group” [24]. This commentary concluded, “The LRC-CPPT has given a new respectability and credibility to the dietary and pharmacological management of hypercholesterolemia.”

In the same year in which the LRC-CPPT findings were published, the National Institutes of Health (NIH) convened a panel of experts on diet, cholesterol, and heart disease. The panel published a consensus statement, which was based, in large part, on the LRC-CPPT findings [25]. The NIH panel concluded, “It has been established beyond a reasonable doubt that lowering definitely elevated blood cholesterol levels … will reduce the risk of heart attacks caused by coronary heart disease. This has been demonstrated most conclusively in men with elevated blood cholesterol levels ….”

The NIH consensus panel failed to address the ensuing controversy over numerous irregularities in the LRC-CPPT statistical methodology. Experts in the field objected to multiple aspects of the data analysis, including concerns with a post hoc relaxation of the threshold with which to determine statistical significance and an apparent post hoc change to the primary endpoint analysis [26-30]. Specifically, commentators accused the LRC-CPPT investigators of changing how they analyzed the data because the initial data analysis, based on their prespecified method paper [17], failed to support the hypothesis that cholesterol reduction would reduce CHD events or mortality. According to Kronmal [27], the statistically significant effect of cholestyramine treatment was “based on a change in criteria that apparently took place after analyzing the data.” Similarly, L’Abbe et al. [26] stated that the LRC-CPPT investigators failed “to mention and justify their post-hoc choice of a more liberal α-level.” And Mann [30] wrote the following: “the study managers, after seeing the result, used an inappropriate ‘one-tailed test’.”

**Figure 1:**
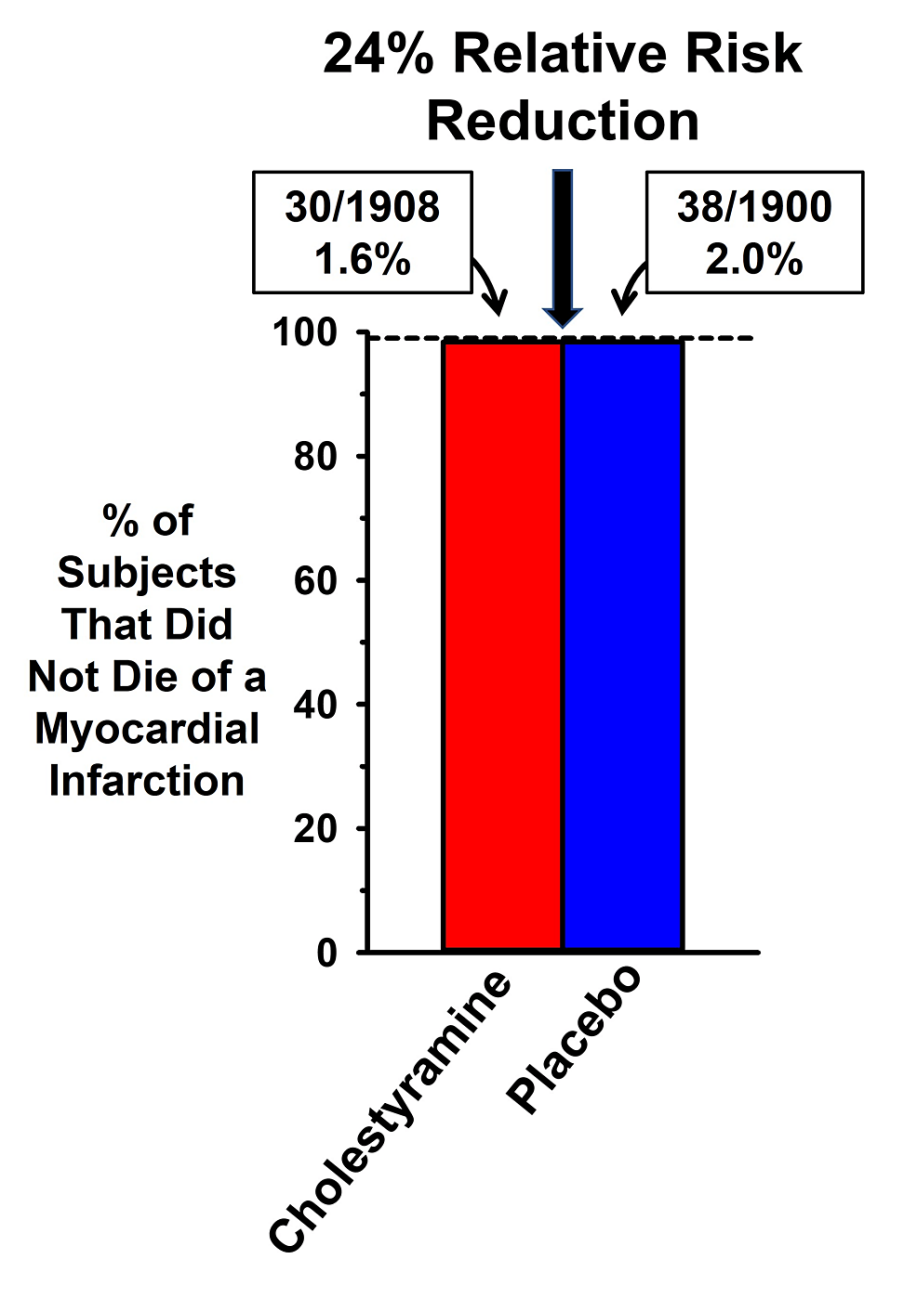
Comparison of absolute and relative risk benefits of cholesterol reduction in LRC-CPPT Data from the Lipid Research Clinics Coronary Primary Prevention Trial (LRC-CPPT) [18]. The 0.4 percentage point absolute risk difference in heart disease mortality between the cholestyramine and placebo groups is actually a 20% relative risk reduction (0.4/2.0) but was converted into a 24% relative risk reduction in events in the LRC-CPPT publication.

A second area of contention involved how the data were portrayed. In Figure 1, we have illustrated the data in the original publication (from their Table 3) to highlight the difference between the magnitudes of the absolute risk reduction and the relative risk reduction. The figure shows that the absolute CHD death rates were 2.0% (38/1,900) for the placebo group and 1.6% (30/1,906) for the experimental group, an absolute difference of 0.4 percentage points. In addition, there were 68 deaths from any cause in the treatment group and 71 in the placebo group (3.7% versus 3.6%), a difference that was not statistically significant.

Praise for the findings of this trial stated that cholesterol reduction resulted in “a 24% reduction in definite CHD death and a 19% reduction in nonfatal myocardial infarction.” How can the CHD mortality rate be reported as a 24% reduction in the publication, as well as in the medical journals that praised the findings, when the difference in CHD mortality between the two groups was only 0.4%? The explanation is that the AR mortality reduction of 0.4% was transformed into an RR reduction by dividing 0.4% by 2.0%, which resulted in a far more impressive 24% effect.

Expressing the data in the RR format provided strong support for the conclusion of the authors that cholesterol reduction was successful at reducing the rate of coronary mortality. The magnitude of this treatment benefit is quantified as the number needed to treat (NNT), which is the inverse of the AR. in this case, the NNT is 250. That is, one would need to treat 250 subjects with this drug for over seven years to delay a single CHD death from occurring, with no effect on overall mortality.

The same RR data transformation was applied to the composite measure of all CHD deaths combined with nonfatal myocardial infarction (MI), which was a 1.6 percentage point difference in events (8.6% {placebo} versus 7% {drug}). The 1.6% (AR) divided by 8.6% results in an RR reduction of 19%. In their summary and in the NIH consensus publication [25], the authors presented only the 19% RR reduction of combined endpoints, without mentioning the AR reduction of 1.6%. It was the 19% RR measure for the composite endpoint, as well as the 24% RR reduction in CHD mortality, that propelled this trial to be the centerpiece of the 1985 NIH consensus report on the benefit of cholesterol reduction for heart health.

Given the negligible effects of cholesterol reduction on coronary events and mortality, LRC-CPPT could well have been dismissed by the steering committee as a negative trial. Instead, the trial directors focused only on the RR, which resulted in the study findings promoted on the cover of TIME magazine [31], with the message that “the cholestyramine group … suffered 19% fewer heart attacks. Their cardiac death rate was a remarkable 24% lower than that of the placebo group.” The Time magazine article quoted Project Director Basil Rifkind, who stated that the LRC-CPPT findings are “a turning point in cholesterol-heart-disease research.”

More objective assessments of flaws with the LRC-CPPT and the NIH consensus panel were provided at the time by two acknowledged experts. The first was from Dr. Thomas Chalmers of Mount Sinai Medical School, who commented, “I think they made an unconscionable exaggeration of all the data” [32]. The second was from Dr. George Mann, professor of biochemistry and medicine at Vanderbilt University Medical School [30]: “They have held repeated press conferences bragging about this cataclysmic breakthrough which the study directors claim shows that lowering cholesterol lowers the frequency of coronary disease. They have manipulated the data to reach the wrong conclusion. This is plain for any student of elementary statistics to see. The managers at NIH have used Madison Avenue hype to sell this failed trial in the way media people sell an underarm deodorant. The (NIH Consensus Panel) has failed to acknowledge that the LRC trial, like so many before it, is saying firmly and loudly ‘No, … the drug you generously tested for a pharmaceutical house does not work …’.”

MRFIT: Miscommunication of risk in the portrayal of CHD mortality

MRFIT had two components, an interventional trial [33] and an observational study [19]. We have focused on the observational component, which assessed the hypothesis that there would be a positive association between serum cholesterol and CHD deaths in middle-aged males. Its independent variable was total cholesterol and not LDL cholesterol (LDL-C). Despite the flaw that total cholesterol is of lesser value in assessing CHD risk [34-39], MRFIT was promoted as demonstrating that small increments, across the entire physiological range of total cholesterol, increased one’s risk of dying of CHD.

MRFIT involved the screening of 356,222 American males, 35-57 years of age, initially free of CHD. The authors reported that they found a “continuous, graded, strong relationship between serum cholesterol and six-year age-adjusted CHD.” The study concluded that “of all CHD deaths, 46% were estimated to be excess deaths attributed (solely) to serum cholesterol 180 mg/dl or greater.”

**Figure 2:**
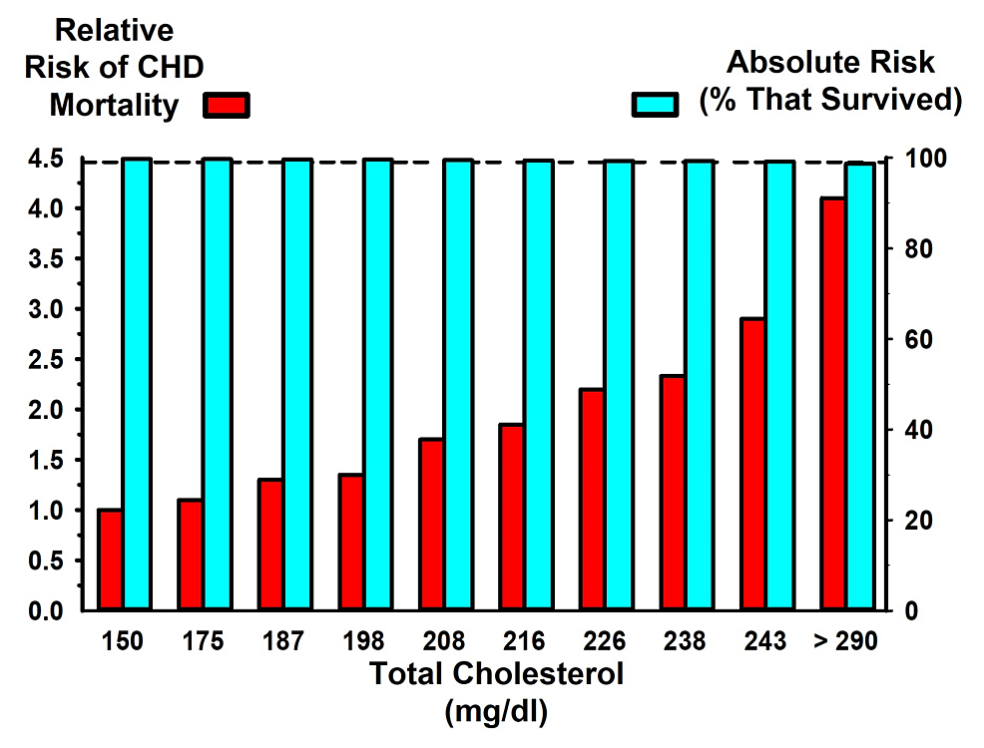
MRFIT findings expressed as the relative and absolute risk of CHD Data from the Multiple Risk Factor Intervention Trial (MRFIT) trial [19] expressed as relative risk (red bars, left y-axis) and as absolute risk (blue bars, right y-axis) CHD: coronary heart disease

The data from Table 3 of the original publication are provided here as Figure 2, which illustrates the relation between serum cholesterol levels (in deciles) and CHD mortality. The same raw data generated the RR (red bars) and the AR (blue bars) for CHD death rates. The data presented as RR seem alarming, since they appear to strongly support the authors’ conclusion that small increments in cholesterol are associated with substantial increases in CHD risk. The graph illustrates that people with total cholesterol levels of 216 mg/dl, 246 mg/dl, and >290 mg/dl have two, three, and four times the risk, respectively, of dying of CHD, compared to people whose total cholesterol level is 150 mg/dl. This dramatic increase in CHD death supported the authors’ conclusion that cholesterol levels above 180 mg/dl “powerfully affects risk for the great majority of middle-aged American men.”

A less alarming view of the MRFIT findings is provided by the overlapping AR data in Figure 2. The blue bars illustrate the AR of CHD in terms of the rate of survival in relation to cholesterol levels (the dashed line provides a visual guide at 99%, which indicates that at every level of cholesterol, the survival rate was approximately 99%). Thus, at the lowest level of cholesterol, CHD survival was 99.7%, and at the highest level of cholesterol, CHD survival was 98.7%. Thus, across the entire physiological range of cholesterol levels, the difference in the rate of six-year CHD mortality was only about one percentage point.

The difference in CHD mortality across the physiological range of cholesterol levels was notably less for the nonsmoking, non-hypertensive subgroup (45% of all subjects). Only 0.3% of all the subjects in this subgroup died of CHD. The AR of CHD death in this group from the highest to lowest cholesterol levels was only 0.48 percentage points (0.64% versus 0.16%). Despite the absence of a clinically relevant AR relation between cholesterol levels and CHD mortality in this subgroup, the authors reported that CHD mortality was four times greater for those with the highest cholesterol compared to those with the lowest cholesterol. Again, the authors reported their findings as the RR (0.64% is four times greater than 0.16%), without mention of the AR, to support their conclusion that elevated levels of cholesterol are implicated “in the causation of premature CHD.”

To put the MRFIT findings into historical perspective, by the 1980s, the hypothesis that cholesterol caused heart disease was falling out of favor. Clinical trials of different agents, such as corn oil [40], clofibrate [41], and cholestyramine [18], had all failed to demonstrate a significant clinical benefit of cholesterol reduction on coronary events and mortality. Had the trial directors focused on AR instead of RR, MRFIT should have served as the death knell for the cholesterol hypothesis. Cardiovascular research would have shifted its focus in subsequent decades on more traditional CHD risk factors, including smoking, stress, hypertension, hyperglycemia, and insulin resistance. Instead, MRFIT, as well as LRC-CPPT, provided the impetus for the expansion of cardiovascular disease (CVD) treatments to new approaches to reduce cholesterol, including the development of statins.

JUPITER: Miscommunication of lipid-lowering benefits in the statin era

Statins reduce cholesterol levels by blocking the activity of 3-hydroxy-3-methylglutaryl coenzyme A (HMG-CoA) reductase, the rate-controlling enzyme in cholesterol synthesis. Statins have been considered so successful in preventing coronary events that William Roberts, MD, editor of the American Journal of Cardiology, described statins as “miracle drugs,” which “are to atherosclerosis what penicillin was to infectious diseases” [42]. Praise for statins has been so strong that critics have been labeled as members of a “statin denial cult” [43], who disseminate “fake medical news and fearmongering … through relentless attacks on statins” [44].

We suggest that statins are promoted as “miracle drugs” largely because statin advocates have often publicized their RR reduction while failing to highlight their modest AR reduction. Moreover, the adverse effects of statins have been minimized or ignored entirely. Diamond and Ravnskov [45] provided a critique of this strategy in their assessment of clinical trial outcomes for statin treatment in primary and secondary prevention of CHD.

Here, we have focused on how the findings of the JUPITER trial were presented in the medical literature and to the public. JUPITER included nearly 18,000 subjects, which was designed to test the hypothesis that treatment with rosuvastatin for primary prevention would reduce vascular events in relatively healthy people with high-sensitivity C-reactive protein (CRP) but without hyperlipidemia [20].

JUPITER was a landmark trial with findings that were embraced by many healthcare providers because it appeared to demonstrate substantial benefits for people without high cholesterol and with a low risk for CHD. In two representative comments on JUPITER outcomes, Dr. Steven E. Nissen, director of cardiology at the Cleveland Clinic, proclaimed, “It’s a breathtaking study. It’s a blockbuster. It’s absolutely paradigm-shifting,” and Dr. W. Douglas Weaver, president of the American College of Cardiology, was equally emphatic: “This takes prevention to a whole new level. Yesterday you would not have used a statin for a patient whose cholesterol was normal. Today you will” [46]. The JUPITER findings appeared so impressive that the trial was terminated prematurely on an ethical basis because in only 1.9 years, there was “a 44% reduction in the trial primary end point of all vascular events, a 54% reduction in myocardial infarction, a 48% reduction in stroke, a 46% reduction in need for arterial revascularization, and a 20% reduction in all-cause mortality” [47].

The finding that medication can reduce the likelihood of a coronary event by 50% may well seem like a miracle treatment, as it would appear that half of all statin-treated people are protected from having a heart attack. However, as with LRC-CPPT and MRFIT, the magnitude of benefits with cholesterol reduction in the original publication [20], as well as in the summary by Ridker [47], emphasized the RR benefit, with little to no mention of the AR.

**Figure 3:**
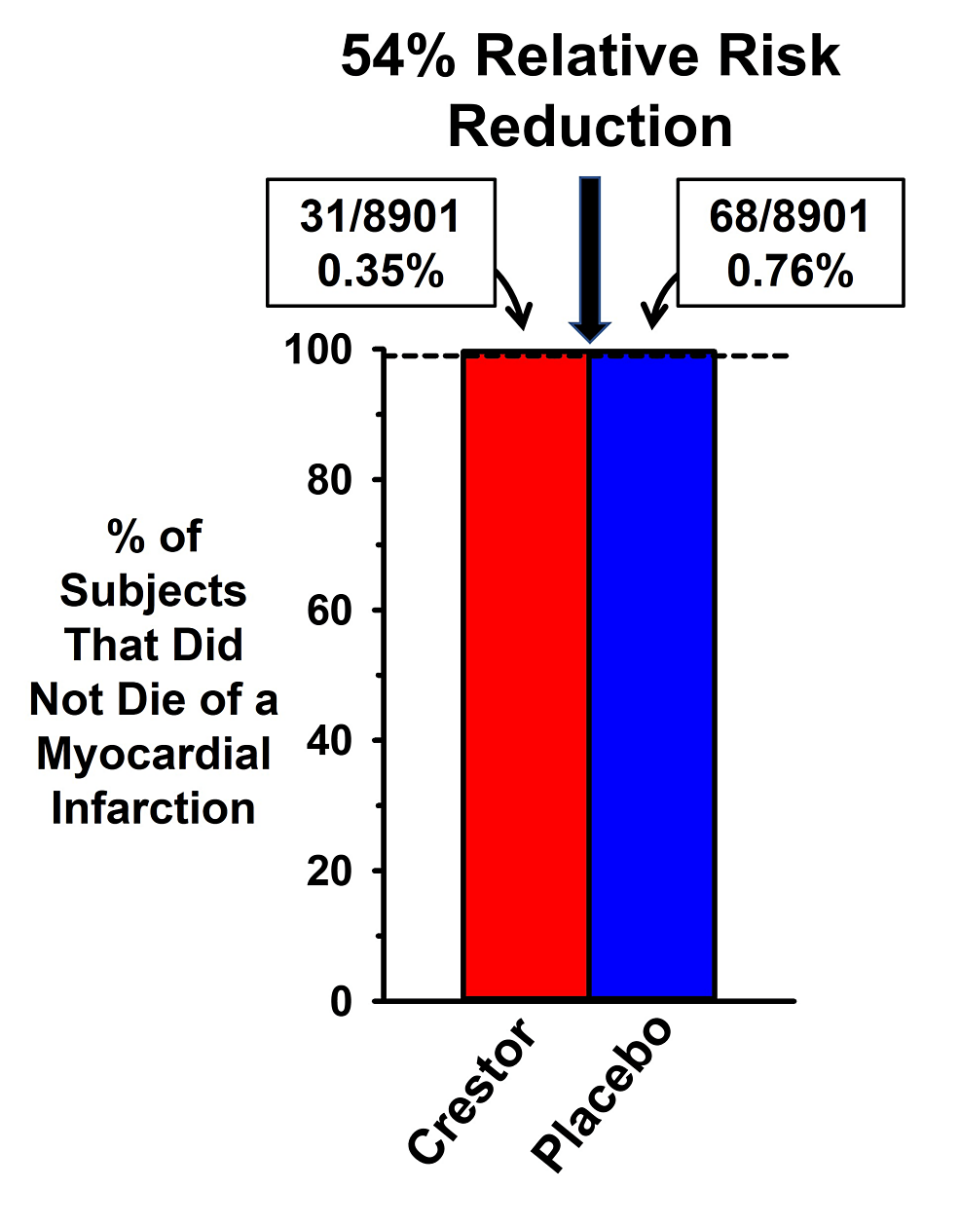
Comparison of absolute and relative risk benefits of cholesterol reduction in the JUPITER RCT Data from the “Justification for the Use of Statins in Prevention: An Intervention Trial Evaluating Rosuvastatin” (JUPITER) randomized controlled trial (RCT) [20], expressed as the percent of subjects in the rosuvastatin group (Crestor) compared to the placebo group without myocardial infarction (MI) mortality. The 0.41 percentage point absolute risk difference in MI mortality between the Crestor and placebo groups was converted into a 54% relative risk reduction in events

Figure 3 is based on the data in Table 3 of the original publication, which illustrates the great disparity between how the AR and RR represent the magnitude of rosuvastatin effects on coronary events. The dashed line at 99% is a visual aid to illustrate that over 99% of rosuvastatin (Crestor) and placebo subjects did not suffer from a fatal myocardial infarction (MI). If more than 99% of all subjects did not die as a result of an MI, how was it that the authors could claim that rosuvastatin reduced the rate of a fatal MI by 54%? As with the LRC-CPPT and MRFIT trials, the data in JUPITER were presented as the RR. The incidence of a fatal MI occurred in 0.76% of the subjects in the placebo group and in 0.35% of the rosuvastatin-treated subjects. Thus, the AR reduction was only 0.41 percentage points, but 0.41 is 54% of 0.76%. It is difficult to understand how an improvement in coronary outcomes in less than one-half of 1% of subjects treated with rosuvastatin generated such unbridled enthusiasm by experts in the field.

Although the AR reduction with rosuvastatin treatment was modest, a small benefit could be of value if statins had very few and only minor adverse effects. However, the adverse effects of statins are extensive [45,48-59], including an increased risk of new-onset type 2 diabetes [20,60-65], an increase in fasting blood glucose in patients with and without diabetes [66], mitochondrial dysfunction [67-69], tendinopathy [70], myopathy [71,72], acute kidney injury/renal failure [73-75], and cognitive deficits [54,76-83].

The JUPITER trial documented a significant increase in the incidence of new-onset diabetes with rosuvastatin treatment compared to placebo (their Table 4). In reporting this adverse effect of statins, the authors presented the data only in terms of its AR, without transforming it into the RR. According to Gigerenzer et al. [84], the portrayal of the RR without including the AR provides “incomplete and misleading information,” which they referred to as “mismatched framing.”

Had the authors of the JUPITER trial used the RR, they would have reported a statistically significant 25% increase in new-onset diabetes with rosuvastatin treatment. In addition to the fact that the authors did not report the diabetogenic effect in terms of its RR, they downplayed its clinical relevance in the original publication, as well as in subsequent work. In a paper addressing the controversy over JUPITER, the trial director considered the increase in diabetes as only a “play of chance” [47]. However, the significant increase in the incidence of diabetes with statin treatment has been reported in numerous subsequent publications [60-64,85], including an RCT that characterized the mechanistic basis as to how statins increase the susceptibility of users to develop diabetes [65]. This finding is relevant to why RCTs have demonstrated an increase in fasting blood glucose in patients with and without diabetes [66], as well as a meta-analysis that suggested females are more susceptible than males to develop type 2 diabetes with statin treatment [60].

The findings of the JUPITER trial were subject to widespread criticism, similar to the controversy generated in response to LRC-CPPT. According to Curtiss and Fairman [86], “Criticism of the JUPITER trial results began immediately” and developed into “an avalanche” of published critiques [87-90]. Criticisms were raised at multiple levels, including questionable justification for terminating JUPITER in less than two years despite the plan to run the trial for four years. There were also concerns that the premature termination was influenced by the pharmaceutical company sponsoring the trial. There was also criticism of the efforts by the authors to downplay the increased incidence of diabetes with drug treatment. It is noteworthy that Serebruany [91] asserted that “the medical community does not uniformly accept the results of the JUPITER trial, engaging in heavy, somewhat personal debates over the validity of the JUPITER findings” including “commercial interests of the principal investigator” and a “fundamental problem with trial integrity.”

Another criticism of JUPITER is also the focus of our commentary. Critics commented that the study outcomes emphasized the RR and ignored the AR reduction. For example, Vaccarino et al. [90] expressed concerns regarding the lack of attention to the modest AR benefit in JUPITER. These authors stated that the trial was terminated prematurely based on the 44% RR reduction in events, but they echoed the concerns of others by stating, “what really matters for dictating changes in clinical practice is the absolute risk reduction.” According to Vaccarino et al., the AR for unambiguous hard events (nonfatal and fatal MI) was just 0.20% per year, which translates into “500 persons need to be treated for 1 year to prevent 1 event.” Despite the appearance of an impressive 54% RR, the modest AR led Vaccarino et al. to conclude that “the treatment benefits achieved in the JUPITER trial are not large enough to advocate an expansion in the clinical indications for statins.”

Nevertheless, despite the unimpressive benefits and evidence of a diabetogenic effect, the FDA granted approval for the use of rosuvastatin for the primary prevention of heart disease in people without high cholesterol but with high CRP and otherwise at a low risk for CHD.

FOURIER and CLEAR: Miscommunication of lipid-lowering benefits in contemporary research

At the American College of Cardiology meeting in 2017, Dr. Marc Sabatine, the principal investigator of the FOURIER trial, was extremely positive in reporting the findings that assessed coronary event outcomes with evolocumab treatment. Unlike statins, which block cholesterol production, evolocumab reduces LDL-C by inhibiting the activity of proprotein convertase subtilisin/kexin type 9 (PCSK9), which is an LDL receptor (LDL-R)-degrading enzyme [92]. In FOURIER, evolocumab reduced LDL-C by 59%, achieving one of the lowest levels of LDL-C reported in any RCT [21].

According to Sabatine, “Evolocumab reduced the risk of cardiovascular events, a 15% reduction in the primary endpoint, a 20% reduction in the risk of cardiovascular death, MI or stroke” [92]. The 15%-20% benefits of evolocumab treatment were highlighted, as well, in the opening paragraph of the Discussion section of the publication [21].

Sabatine’s approach to data presentation followed the now routine practice of mentioning only the RR, without including the AR. The impact of the FOURIER findings may have been less impressive had the reduction in the risk of the composite of cardiovascular death, MI, or stroke been expressed as the AR, which was only a 1.5 percentage point difference between treatment and placebo (5.9% versus 7.4%).

It is also notable that the 20% benefit that Sabatine mentioned and stated in the Discussion section of the publication was based on a composite of heterogeneous outcomes in multiple CVD categories, which included significant, as well as non-significant, effects. Of clinical importance is the finding that there was no statistically significant effect on CVD death (or death due to MI, stroke, or “other CVD death”). In addition, there was no effect of treatment on all-cause mortality.

Of the three CVD event categories in the composite measure reported to have a 20% benefit (CVD death, MI, or stroke), only one category exhibited an AR benefit greater than a single percentage point; there was a 0.4% AR reduction in the incidence of stroke (1.9% versus 1.5%), a non-significant 0.1% increase in the incidence of CVD death (1.7% versus 1.8%), and a 1.2% AR reduction in the incidence of MI (4.6% versus 3.4%), placebo versus drug, respectively. Thus, the 20% RR and 1.5% AR reduction in the composite measure of CVD death, MI, or stroke was carried almost entirely by the 1.2% AR in MI.

A recently published paper provided an update on long-term effects of evolocumab treatment on coronary event rate [93]. In the Clinical Perspective section of the paper, which highlighted the most important findings of the study, the authors stated, “patients who were originally randomized to evolocumab had a 15-20% lower risk of major adverse cardiovascular events and 23% lower risk of cardiovascular death than those randomized to placebo.” As before, the RR reduction was highlighted, and the AR reduction was not mentioned in the paper.

The reporting of the FOURIER findings in reviews followed the established practice of portraying the benefits of lipid-lowering therapy in the RR format, without addressing the AR. In one example, Libby and Tokgözoğlu [94] stated that evolocumab reduced cardiovascular events by 15%-20%, without mentioning the AR. Their review failed to note that there were no statistically significant effects of evolocumab treatment on a range of CVD measures, including CVD death or hospitalization from heart failure, death from any cause, or hospitalization for unstable angina. In fact, the 1.2 percentage point difference in AR reduction of MI (from 4.6% to 3.4%, placebo versus treatment, respectively) was the only hard event category, that is, MI, death, and stroke, of 13 individual categories, in which the AR difference between evolocumab and placebo exceeded 0.5 percentage points. Despite the negligible overall benefit of evolocumab on most CVD outcomes, Libby and Tokgözoğlu [94] concluded that PCSK9 medication serves as a model “in the quest to conquer cardiovascular diseases.”

In the most recent RCT on lipid-lowering therapy, statin-intolerant patients were administered a placebo or bempedoic acid [22], which is similar to statins in that it reduces hepatic cholesterol synthesis and raises LDL receptor expression, thereby increasing the clearance of LDL cholesterol from the circulation. Unlike statins, bempedoic acid is activated in the liver and not in most peripheral tissues, including skeletal muscle. Bempedoic acid, therefore, was expected to have less of an adverse effect on muscles than statins.

The group-administered bempedoic acid exhibited a 26.1% reduction in LDL-C levels (compared to a 10.6% reduction in the placebo group). The AR reduction with bempedoic acid for combined major coronary adverse events was 1.5 percentage points (13.3% versus 11.7%), with no benefit in fatal or nonfatal stroke or death from any cause, including cardiovascular causes. Bempedoic acid treatment produced adverse effects not seen with statins, including a significant increase in hyperuricemia (10.9% versus 5.6%), gout (3.1% versus 2.1%) and cholelithiasis (gall stones) (2.2% versus 1.2%), renal impairment (11.5% versus. 8.6%), and elevated hepatic enzyme level (4.5% versus. 3.0%).

In a discussion of the CLEAR outcomes, Dr. Ann Marie Navar did not mention the AR 1.6 percentage point difference in the benefits of bempedoic acid treatment. Instead, she presented the benefits of bempedoic acid in terms of the RR data, by stating that there was a 13% RR reduction in events [95]. In contrast, when Dr. Navar addressed the adverse effects of the treatment, she mentioned only the AR data, stating that there was only a 1% increase in the incidence of gout and cholelithiasis (the formation of gallstones), with no mention of the increased incidence of renal impairment, elevated hepatic enzyme level, and near doubling of the incidence of hyperuricemia with bempedoic acid treatment versus placebo. Gigerenzer et al. [84] considered this form of data presentation to be the second “sin” against transparent reporting, that is, deliberately reporting benefits as relative risk reductions while reporting harms as absolute risk increases.

Our perspective

We are not the first to object to the preferential use of RR, without sufficient attention to AR, in clinical trial reporting. For decades, academicians have deplored the strategy taken by some clinicians to promote their findings by emphasizing RR to the exclusion of reporting the AR [6-16]. In one example, Gigerenzer et al. [84] considered this form of data presentation to be “the first ‘sin’ against transparent reporting.”

We have considered two consequences of the preferential use of RR, without sufficient attention to AR, in clinical trial reporting and in the perceived risk of elevated LDL-C. First, the emphasis on promoting relative risk benefits in different areas of clinical trial findings appears to have become an accepted practice in major medical journals. In a study of bias toward presenting RR in favor over AR, Schwartz et al. [96] reported that in a five-year review of four medical journals (Lancet, BMJ, Journal of the American Medical Association (JAMA), and NEJM), 68% of the papers in these journals reported only the RR in the abstracts and only about 50% reported the AR in the entire paper. These authors concluded that the focus on RR and the exclusion of AR in publications “can easily exaggerate readers’ perceptions of benefit or harm” (of a treatment).

As an example of how healthcare providers can be misled by an exclusive presentation of data as RR, Bucher et al. [97] demonstrated that physicians were more inclined to prescribe cholesterol-lowering medication for hypercholesterolemia when the trial results for identical endpoints were expressed as RR reduction, compared to AR reduction. Sackett and Cook [98] commented on this finding by stating that “restricting the reporting of efficacy to just relative risk reductions can lead to greater - and at times - excessive zeal in decisions about treatment for patients with low susceptibilities.”

The bias of physicians toward prescribing lipid-lowering medication, as well as lay people accepting treatment, when trial results were reported solely in the RR format was addressed empirically by Bobbio et al. [11] and Hux and Naylor [9]. In these two studies, the investigators presented real-world data from a lipid-lowering RCT (the Helsinki Heart Study [99]) to physicians [11] and to patients [9]. The subjects of both studies were informed of the AR (a 1.4% difference in cardiac events between treated and untreated individuals) and the RR (a 34% cardiac event reduction) of a treatment. They were then asked if they would prescribe the medication (in the physician study [11]) or if they would take the medication (in the patient study [9]). Unbeknownst to the subjects of both studies, the AR and RR were based on the same raw data (coronary events occurred in 4.1% of the subjects in the placebo group and in 2.7% of the subjects in the lipid-lowering group; an AR reduction of 1.4% and an RR reduction of 34%).

**Figure 4:**
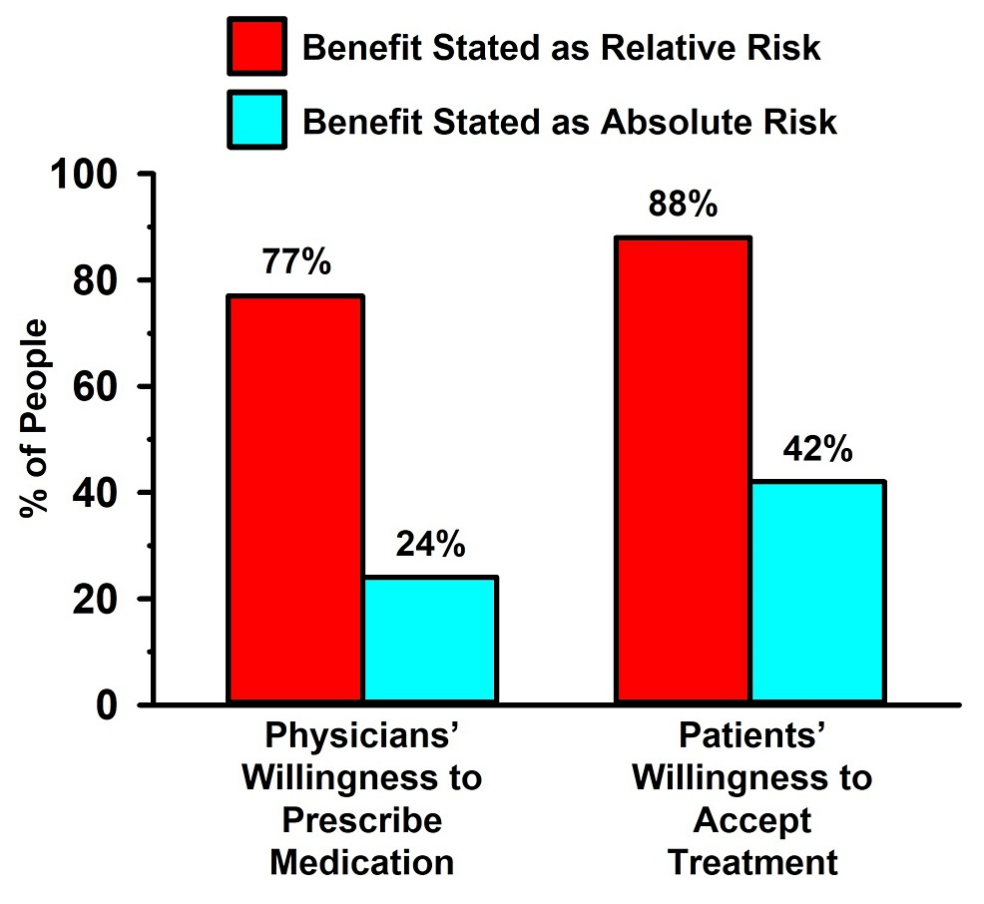
Influence of data presented as relative risk versus absolute risk in patient and physician decision-making Data from Bobbio et al. [11] (left) and Hux and Naylor [9] (right) trials

The findings of the Bobbio et al. [11] and Hux and Naylor [9] studies are illustrated in Figure 4. When the lipid-lowering effects were presented as RR, more than three-quarters of the clinicians were amenable to prescribing the medication, and even more of the patients were amenable to taking the medication. By contrast, when the data were presented as AR, only a quarter of the physicians were amenable to prescribing the medication, and less than half of the patients were amenable to taking the medication. The findings of this study, as well as others with similar outcomes [6-16,100-102], illustrate how presenting RCT outcomes in the AR versus the RR form can influence people as to their confidence in the effectiveness of an intervention.

Stegenga [103] emphasized that “Effectiveness always should be measured and reported in absolute terms (using measures such as ‘absolute risk reduction’)” and he further stated that “people’s comparative understanding of relative versus absolute outcome measures is dubious. Relative measures … fundamentally mislead patients into overestimating effectiveness.” Nevertheless, peer-reviewed medical publications and the media exhibit a high prevalence of bias toward reporting the RR and ignoring the AR.

In our view, this strategy by clinical trial investigators to amplify the appearance of the magnitude of their intervention is unacceptable scientific behavior. We concur with Gigerenzer et al. [84] that journal editors should enforce transparent reporting of data, including the requirement that papers include the AR and RR in the abstracts. In addition, an education on the miscommunication of risk and benefits in research should be included in the training of healthcare workers and students of public health.

Second, we suggest that the exaggeration of the putative harms caused by elevated LDL-C [104] and the benefits of lipid-lowering medications has detracted from an appreciation of the physiological relevance of elevated levels of LDL-C in optimal health. For example, LDL-C is an important component of the immune system [105-107]. Chronically elevated LDL-C levels may enhance aspects of immune functioning, which is potentially relevant to the finding that elderly people with familial hypercholesterolemia (FH) have lower rates of mortality from cancer and infection compared to the general population [108-110].

The importance of LDL-C to overall health may explain why LDL-C is such a poor marker of risk for CVD [34-38], as well as cardiovascular and all-cause mortality [39]. Coronary artery calcification (CAC), in contrast to LDL-C, is the single best predictor of future fatal and nonfatal coronary events [111-121]. Moreover, among those with genetically confirmed FH, approximately half showed no detectable CAC and had a favorable prognosis, despite significantly elevated LDL-C levels [122]. These observations help to explain why FH individuals do not face an increased risk of CVD mortality with advanced age, as well as the greater longevity of people in the general population with high LDL-C, compared to those with low LDL-C [39].

It is also important to note that what is often overlooked in the discussion about LDL-C as a cardiovascular risk factor is the heterogeneity of different LDL particles. That is, the “total LDL-C” reported in a conventional lipid panel represents the sum of a heterogeneous population of different low-density lipoprotein particles [38]. Specifically, LDL-C is contained in heterogeneous particles that range in size and composition from a small, dense LDL (sdLDL) to a large, buoyant LDL (lbLDL). Circulating sdLDL, unlike lbLDL, readily undergoes atherogenic modifications in plasma, including glycation, which is associated with heightened inflammation, hyperglycemia, insulin resistance, and an increased incidence of CVD in the general population [123-126] and in FH individuals [127-131].

Finally, the overestimation of the involvement of LDL-C in producing CHD based on RR statistics is confirmed by the modest AR benefits of statins, as reflected by the substantial number needed to treat (NNT), that is, the number of individuals that needed to be treated to prevent one event from occurring. This finding was quantified recently by Byrne et al. [132] in a systematic review and meta-analysis of statin RCTs. These investigators reported that the AR reduction of statins was only 0.6% for all-cause mortality, 0.7% for MI, and 0.3% for stroke and 0.9%, 2.2%, and 0.7%, respectively, in primary and secondary prevention. These modest AR benefits of statin therapy yield NNTs of 50-200 to reduce the occurrence of a single coronary event in an average of 4.4 years of treatment. This finding of limited benefits of statins is further confirmed by the work of Kristensen et al. [133] who reported that overall, statin treatment delayed death in primary and secondary prevention trials by only 3.2 and 4.1 days, respectively. These findings support the conclusions of Byrne et al. [132] that “when considering the ARR of statins, the benefits are quite modest, and most trial participants who took statins derived no clinical benefit.”

Hence, the pejorative view of LDL-C as the “bad cholesterol,” which has been perpetuated by the disproportionate emphasis on RR statistics, is not supported by a balanced review of the literature. The characteristic of this perspective is the opinion that “evidence falsifying the hypothesis that LDL drives atherosclerosis has been largely ignored” [134], and the opinion of three cardiologists that “LDL cholesterol risk has been exaggerated” [135] (see also Ravnskov et al. [34] and Diamond et al. [136] for related reviews and discussion).

## Conclusions

We have reviewed the findings of five landmark clinical trials conducted over the past four decades that have supported the current consensus that high LDL cholesterol causes CVD and that the pharmacological reduction of LDL produces substantial CVD benefits. Our assessment of these trials demonstrates that the association of cholesterol with CVD is far more modest than has been portrayed. We also assert that the promotion of statins as “miracle drugs” has been based on an emphasis on their RR reduction, which amplifies the appearance of their modest AR benefits.

The biased approach to data analysis in the five trials we have reviewed is representative of the now common practice of highlighting RR over AR in data presentation and in the media, which has been referred to as a “miscommunication of risk.” A consequence of this biased approach to the reporting of CVD clinical trial findings explains in large part why healthcare providers and the public have overestimated the purported hazards of high cholesterol and the benefits of cholesterol reduction.

In conclusion, for the past four decades, academics have repeatedly asserted that the portrayal of clinical trial findings as the RR, while disregarding the AR, is a deceptive practice. In the cardiovascular disease field, a consequence of this strategy has resulted in the exaggerated appearance of the purported hazards of high cholesterol and an amplification of the magnitude of benefits of cholesterol-lowering medications. This strategy appears to have been deployed for the first time with the publication of the LRC-CPPT trial in 1984 but has now become commonplace. To counter this trend of scientific misconduct, we assert that publications and media reports of clinical trial findings should always portray the benefits, as well as harms, of interventions in terms of both absolute and relative risks.
