# Correction: Historical Review of the Use of Relative Risk Statistics in the Portrayal of the Purported Hazards of High LDL Cholesterol and the Benefits of Lipid-Lowering Therapy

**DOI:** 10.7759/cureus.c116

**Published:** 2023-05-17

**Authors:** David M Diamond, Paul E Leaverton

**Affiliations:** 1 Psychology, University of South Florida, Tampa, USA; 2 Epidemiology and Biostatistics, University of South Florida, Tampa, USA

This article has been corrected at the request of the authors to change Figure 1 to an updated version. In the initial published version of this article the data in the cholestyramine and placebo boxes were reversed. The authors deeply regret that this error was not identified and addressed prior to publication.

